# Ion Mobility Spectrometry after Chromatography—Accomplishments, Goals, Challenges

**DOI:** 10.6028/jres.093.103

**Published:** 1988-06-01

**Authors:** Herbert H. Hill, Randy L. Eatherton

**Affiliations:** Department of Chemistry, Washington State University, Pullman, WA 99164-4630

## The Promises

Ion mobility spectrometry was first introduced to the analytical community in 1970 as an ultratrace organic technique under the name of plasma chromatography. Using highly sensitive ionization methods commonly employed in electron capture detectors coupled with an atmospheric pressure drift region to separate ions produced, it had the potential for widespread application in the separation and detection of trace quantities of organics.

## The Problems

As researchers began to investigate the method, a number of serious problems rapidly became evident: Competitive ion-molecule reactions which occurred in the ionization region of the detector limited its use as a quantitative instrument when complex mixtures were introduced into the spectrometer. Long residence times (sometimes measured in hours) in the spectrometer prevented practical application to a large number of samples. Often spectra for a single component were found to contain multiple ion peaks, hopelessly complicating true multicomponent interpretation.

## Recent Accomplishments

We designed and constructed an ion mobility spectrometer that was specifically suited for the detection of trace organics after separation by high resolution gas chromatography [1]. Using a unique unidirectional flow design, problems associated with long residence times in the spectrometer were eliminated. The high resolution gas chromatograph insured the purity of standards and enabled the introduction of sufficiently small quantities of compounds such that only simple uncomplicated spectra were observed by the ion mobility spectrometer. Microprocessor-controlled operation of the ion gates in the drift tube permitted both selective and nonselective detection of chromatographic effluent [2] and the Fourier transform method of operation enabled spectra from rapidly eluting capillary chromatographic peaks to be captured “on the fly” [3]. It was also found that the linear range of the detector could be extended by the use of a photoionization source [4]. As a GC detector the IMD has been compared to the FID and the ECD for contamination effects [5] and has been applied to the detection of 2,4-D in soils [6] and for the determination of barbiturates [7].

## Current Goals

More recently, our work has led to investigation of the mobility of ions produced from organic compounds with molecular weights higher than those that can be conveniently eluted from a gas chromatograph. One approach to achieve this goal has been to use supercritical fluid chromatography as the introduction method for IMS [8,9]. Besides the detection of medium range (500–5000 amu) molecular weight compounds, advantages of ion mobility detection after SFC include sensitive detection of compounds which do not contain chromophores, universal detection mode of operation, tunable selective detection, Fourier transform spectra of SFC separated compounds, and compatibility with a wide range of mobile phases.

## Future Challenges

Now that interfaces and operational modes for the ion mobility detector have been developed for both gas and supercritical fluid chromatography, the methodology is being investigated for application to real samples. The real challenge for ion mobility spectrometry as a chromatographic detector, however, lies in the detection of compounds separated by liquid chromatography. A significant step toward achieving this goal is the recent development of an electrospray ion source for IMS. By introducing the effluent from a liquid stream into the spectrometer via an electrically charged capillary, we have been able to capture ion mobility spectra from nonvolatile compounds dissolved in the mobile phase. The practical utility of this method of ionization is currently under investigation.
